# Integrase inhibitors versus efavirenz combination antiretroviral therapies for TB/HIV coinfection: a meta-analysis of randomized controlled trials

**DOI:** 10.1186/s12981-021-00348-w

**Published:** 2021-05-01

**Authors:** Yuanlu Shu, Ziwei Deng, Hongqiang Wang, Yi Chen, Lijialong Yuan, Ye Deng, Xiaojun Tu, Xiang Zhao, Zhihua Shi, Minjiang Huang, Chengfeng Qiu

**Affiliations:** 1 Department of Evidence-Based Medicine and Clinical Center, The First People’s Hospital of Huaihua, University of South China, Huaihua, 418000 People’s Republic of China; 2 Department of Clinical Pharmacy, The First People’s Hospital of Huaihua, University of South China, Huaihua, 418000 People’s Republic of China; 3 Department of Intensive Care Unit, The First People’s Hospital of Huaihua, University of South China, Huaihua, 418000 People’s Republic of China; 4 Department of General Practice, The First People’s Hospital of Huaihua, University of South China, Huaihua, 418000 People’s Republic of China; 5 Hunan University of Medicine, Huaihua, 418000 People’s Republic of China

**Keywords:** Integrase inhibitors, Efavirenz, Raltegravir, Dolutegravir, TB, HIV patients

## Abstract

**Background:**

Integrase inhibitors (INIs)-based antiretroviral therapies (ART) are more recommended than efavirenz (EFV)-based ART for people living with HIV/AIDS (PLWHA). Yet, the advantage of integrase inhibitors in treating TB/HIV coinfection is uncertain. Therefore, the objective of this systematic review is to evaluate the effects and safety of INIs- versus EFV-based ART in TB/HIV coinfection, and demonstrate the feasibility of the regimens.

**Methods:**

Four electronic databases were systematically searched through September 2020. Fixed-effects models were used to calculate pooled effect size for all outcomes. The primary outcomes were virologic suppression and bacteriology suppression for INIs- versus EFV-based ART. Secondary outcomes included CD4^+^ cell counts change from baseline, adherence and safety.

**Results:**

Three trials (including 672 TB/HIV patients) were eligible. ART combining INIs and EFV had similar effects for all outcomes, with none of the point estimates argued against the INIs-based ART on TB/HIV patients. Compared to EFV-based ART as the reference group, the RR was 0.94 (95% CI 0.85 to 1.05) for virologic suppression, 1.00 (95% CI 0.95 to 1.05) for bacteriology suppression, 0.98 (95% CI 0.95 to 1.01) for adherence. The mean difference in CD4^+^ cell counts increase between the two groups was 14.23 cells/μl (95% CI 0− 6.40 to 34.86). With regard to safety (adverse events, drug-related adverse events, discontinuation for drugs, grade 3–4 adverse events, IRIS (grade 3–4), and death), INIs-based regimen was broadly similar to EFV-based regimens. The analytical results in all sub-analyses of raltegravir- (RAL) and dolutegravir (DTG) -based ART were valid.

**Conclusion:**

This meta-analysis demonstrates similar efficacy and safety of INIs-based ART compared with EFV-based ART. This finding supports INIs-based ART as a first-line treatment in TB/HIV patients. The conclusions presented here still await further validation owing to insufficient data.

**Supplementary Information:**

The online version contains supplementary material available at 10.1186/s12981-021-00348-w.

## Introduction

Tuberculosis (TB) is the most common opportunistic infection in people living with HIV/AIDS (PLWHA) and has significant regional differences, especially in resource-limited countries. TB is also one of the main causes of death of PLWHA [1, 2]. HIV infection increases the risk of progression to active TB disease [3], which challenges the goal of the “90–90–90” targets of HIV by 2020 and the end TB strategy by 2035. A novel strategy recommending a 6 months of isoniazid preventive therapy (IPT) on PLWHA is essential in preventing latent TB progressing into active disease from latent TB, and will reduce both the incidence and subsequently mortality of TB [4].

People with TB/HIV co-infection typically present with low CD4^+^ cell counts and high HIV viral loads. TB/HIV patients require co-treatment for both diseases, in particular for the TB [5]. During this critical period, effective management of the two diseases is essential to improve the survival and quality of life for co-infection patients. However, co-treatment with TB and HIV is challenging, owing to drug interactions, overlapping toxicities, and a high risk of immune reconstitution inflammatory (IRIS) [6]. Among these factors, drug interaction between antiretroviral and antituberculosis greatly counteracts the antiretroviral therapy.

Rifampicin (RIF), the standard TB treatment regimen of four chemical drugs, remains the first choice, irrespective of the patient’s HIV status [5]. Although an increasing number of new antiretroviral drugs are available to treat HIV infection, only a few can be used in patients with co-infection due to the drug-drug interactions. In countries with limited resources and a high burden of TB, efavirenz (EFV) and nevirapine (NVP) are the preferable options for initial antiretroviral treatments (ART) in TB/HIV co-infection [7]. Due to the high rate of virological failure and adverse reactions [8–10], NVP is not recommended as a preferred therapy for the antiviral treatment of TB/HIV patients. While EFV metabolism is affected by certain genetic polymorphisms [11] with high inter-patient variations in plasma concentrations. When combined with RIF, the effect may magnify [12–14]. Some studies recommend increasing the dosage of EFV to counteract the inductive effects of RIF [15, 16], but there is no sufficient evidence to support the efficacy of this approach [14, 17, 18]. Besides, there remain several challenges about EFV, such as EFV-related adverse effects and drug resistance [19, 20]. In the event of contraindications or intolerance to EFV, it is urgent to find a novel alternative agent. Protease inhibitors (PIs) may offer a solution to these problems and even additional advantages [21]. Noteworthy, PIs needs to be rifabutin (RBT) fixed collocation to co-treat TB/HIV, but the optimal RBT dosing frequency is unknown [22, 23]. TB treatment is usually given in fixed-dose combinations, and considering the cost or non-availability of RBT, it is difficult to substitute RBT for RIF in many settings.

Integrase inhibitors (INIs), mainly include raltegravir (RAL) and dolutegravir (DTG), exhibit good efficacy and safety, and are superior to PIs in terms of durability [24]. Since there is no need to add ritonavir boosting like PIs, in high-income countries, RAL and DTG are preferred first-line ART for the treatment-naïve and treatment-experienced PLWHA. Commendably, a breakthrough low price agreement, which provides accessibility drugs in low- and middle-income countries was achieved in 2017 [25]. INIs cite better efficacy, reduce treatment discontinuation, and serve as a higher genetic barrier to resistance than efavirenz-based ART [26]. INIs, the substrate by several metabolic enzymes for UGT1A1 and cytochrome CYP3A, are also affected by RIF induction. When co-administered with RIF, there are substantial reductions in the blood concentration of INIs, and hence doubling the dose of both drugs can overcome the induction by RIF [27, 28]. As a hypothesis, INIs-based ART may be more favorable than EFV-based ART in TB/HIV patients. Currently, little data are available regarding the effect of INIs on TB/HIV patients [29]. There is no clinical evidence to support switching patients from the EFV-based regimen directly to the INIs-based regimen [30]. To provide a best available evidence, and refine the existing clinical guidelines, we collected a limited number of randomized controlled trials (RCTs), compared INIs- and EFV-based ART according to the principles of evidence-based medicine.

## Methods

### Study design

The study followed the Preferred Reporting Items for Systematic Reviews and Meta-Analyses (PRISMA) guidelines [31].

### Data sources and searches

PubMed, Medline, Embase, and Cochrane Library until 17 September 2020 were systematically searched. Studies were limited to the English language. Using the following keywords: ‘tuberculosis’, ‘raltegravir’, ‘dolutegravir’, and ‘integrase inhibitor’. We used the names of two available INIs drugs as keywords, as both drugs are the primarily recommended drugs.

### Study selection and eligibility criteria

RCTs that compared ART in INIs- with EFV-based, in TB/HIV patients with treatment-naïve, and evaluated at least one outcome of effectiveness and/or safety. The INIs included DTG and RAL, while EFV-based ART was the preferred therapeutic regimen. All patients received a standard tuberculosis treatment regimen with isoniazid, rifampicin, pyrazinamide, and ethambutol for the first 2 months, followed by isoniazid and rifampicin for the subsequent 4 months. Rifampicin was given at a dose of 10 mg per kg per day. In case of extrapulmonary TB, the duration of the maintenance regimen was extended. Antiretroviral treatment was started after 2–8 weeks of TB treatment.

We ignored results with a follow-up length shorter than 48 weeks (e.g. 24 weeks), which was not sufficient for evaluating clinically significant outcomes for intervention. The timeframe was applied in the previous systematic reviews on HIV treatment [32]. If the same study were overlapped in multiple publications, only the complete or most recent literature was included in the present study.

After removal of duplicates, all studies identified in the search were screened by title and abstract by two independent reviewers (YS and ZD), then full-texts were reviewed to determine eligibility. All the incongruity was resolved by group discussion or a third reviewer (CQ).

### Data extraction and quality assessment

Data extracted included: (1) research characteristics (author, year of publication, study design, and sample size); (2) patient demographics (age, sex, and race) and baseline characteristics (CD4^+^ cell counts, viral load); and (3) result at the end of the study.

The risk of bias was evaluated the Cochrane Collaboration tool [Cochrance Handbook for Systematic Reviews of Intervention, version 5.1.0]. Assessed risk of bias included six specific domains: sequence generation, allocation concealment, blinding, incomplete data, selection outcome reporting, and other possible biases. The risk of bias in each domain was judged as “low risk”, “high risk”, or “unclear risk”, with the last category bias indicating either lack of information or uncertainty over the potential source of bias.

### Outcomes

#### Efficacy

The primary efficacy outcomes were the percentages of participants with virologic and bacteriology suppression at week 48. The response was assessed using a modified US FDA Snapshot algorithm [33], in which participants with HIV-1 RNA ≥ 50 copies/ml or without HIV-1 RNA data at week 48 were both considered as non-responders. Another primary outcome was TB treatment outcomes according to WHO definitions [34]. The secondary efficacy outcomes were the mean increase in CD4^+^ cell counts from baseline and the ratio of adherence at Week 48.

#### Safety

Safety outcomes included the percentage of participants with adverse events (AEs), drug-related AEs, discontinuation for drugs, grade 3–4 AEs, IRIS (grade 3–4), and death during the 48 weeks.

### Data analysis

EFV-based ART was used as the reference group in the meta-analysis. For continuous outcomes (that is, CD4^+^ cell counts) in this systematic review, for efficient merging, the median and interquartile range (IQR) were converted into mean and standard deviation (SD) [35, 36]. A difference in mean change greater than 0 favored INIs-based ART. For the dichotomous outcomes, a risk ratio (RR) with 95% confidence interval (CI) was calculated based on the number of total participants and the number of events in each group within each study. Then the studies was pooled to obtain an overall effect estimate. A RR greater than 1 favored INIs-based ART for all dichotomous outcomes. To evaluate the consistency of INIs, in studies with predefined subgroups, we only performed subgroup analysis including DTG and RAL.

Statistical heterogeneity among the studies was measured by the Cochrane Q test and I^2^ statistic. To account for the heterogeneity across the studies, the fixed-effect model was used to combine the effect estimate from included studies.

The possibility of publication bias estimated by funnel plots was not performed, because the number of studies included in the meta-analysis was fewer than 10. In such a case, the funnel plots could yield misleading results and therefore were not recommended.

All data analyses were performed with Review Manager 5.4 (Cochrane Collaboration, London, UK).

## Results

**Fig. 1:**
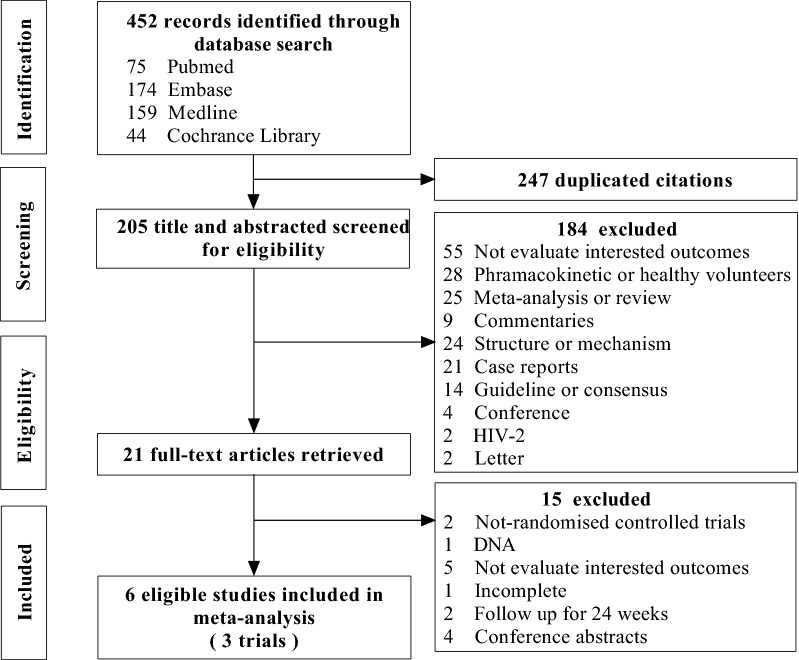
Flow diagram: Study screening process

The flow chart summarized the detailed retrieval steps (Fig. 1). We initially identified 452 articles from four databases. By screening titles, abstracts, and full texts, finally three eligible clinical trials (reported in 3 published articles and 3 clinical trial registration) were included in this meta-analysis.

The main features of the included studies are summarized in Additional file 1: Table S1. 672 treatment-naïve patients receiving a standard TB treatment regimen were randomized in the three trials. In the ANRS 12,180 [37, 38] and ANRS 12,300 [39, 40] trials, RAL plus TDF/3TC was compared with EFV plus TDF/3TC. While in the INSPIRING [41, 42] trial, DTG was compared with EFV plus major TDF/FTC, or plus other two N(t)RTIs. Drug doses of INIs (i.e. DTG and RAL) were double-dosed (twice-daily). All studies' analyses were open-label, randomized, and noninferiority clinical trials. Only one trial did allocation concealment. All trials did not described blinding of outcome assessment. One determine the exception of the blinding of participants and personnel because of the open-label nature of all study design (see Additional file 2: Fig. S1 and Additional file 3: Fig. S2).

### Efficacy

**Fig. 2:**
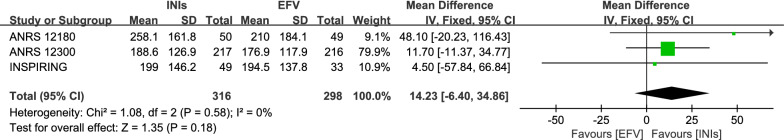
Forest plot of compared CD4^+^ cell counts recovery with the two regimens at 48 weeks

None of the INIs-based group was significantly less than any of the EFV-based group on any of the efficacy outcomes assessed (Table 1). As shown in this Meta-analysis, INIs and EFV groups had similar effects on all critical outcomes, with none of the point estimates argued against the INIs-based ART. In the intent-to-treat (ITT) population, 231 patients on INIs (66%) and 223 patients on EFV (69.3%) had HIV-RNA < 50 copies/ml. The overall RR in the pooled proportion of virologic suppression between two regimens was 0.94 (95% CI 0.85 to 1.05). Bacteriology suppression, another key outcome, showed an overall RR of 1.00 (95% CI 0.95 to 1.05). The conclusion from these two primary efficacy outcomes was consistent. Poor adherence to antiretroviral therapy could contribute to HIV treatment failure, while in both groups, the adherence was high and similar with an overall RR was 0.98 (95% CI 0.95 to 1.01). The mean increase in CD4^+^ cell counts from baseline to Week 48 was 201.2 cells/μl for the INIs group and 184.3 cells/μl for the EFV group [mean difference 14.23 cells/μl (95% CI -6.40 to 34.86), Fig. 2].

**Table 1:** Summary of the pooled efficacy outcome

Outcome or subgroup	Studies	Cases	No. with event/total no. of patients INIs and EFV	Effect estimate (RR and 95% CI)
Virologic suppression	3	672	231/350 and 223/322	0.94 (0.85,1.05)
DTG vs. EFV	1	113	52/69 and 36/44	0.92 (0.76,1.12)
RAL vs. EFV	2	559	179/281 and 187/278	0.95 (0.84,1.07)
Bacteriology suppression	3	672	313/350 and 288/322	1.00 (0.95,1.05)
DTG vs. EFV	1	113	61/69 and 40/44	0.97 (0.86,1.10)
RAL vs. EFV	2	559	252/281 and 248/278	1.01 (0.95,1.06)
Adherence	3	617	309/321 and 291/296	0.98 (0.95,1.01)
DTG vs. EFV	1	99	56/58 and 38/41	1.04 (0.94,1.15)
RAL vs. EFV	2	518	253/263 and 253/255	0.97 (0.94,1.00)

### Safety

The occurrence of all AEs was similar in patients between the two groups (Table 2). Compared to control, the incidence of drug-related AEs of INIs-based group was lower, but these difference was not statistically significant between the two groups. Co-treatment could elicit a serious condition called IRIS in TB/HIV patients. The occurrence of IRIS (grade 3–4) observed in three studies was infrequent. The overall RR was 0.63 (95% CI 0.32 to 1.25) between the two groups.

**Table 2:** Summary of the pooled safety outcome

Outcome or subgroup	Studies	Cases	No. with event/total no. of patients INIs and EFV	Effect estimate (RR and 95% CI)
Any AEs	2	215	98/120 and 86/95	0.92 (0.76,1.11)
DTG vs. EFV	1	113	52/69 and 40/44	0.83 (0.70,0.98)
RAL vs. EFV	1	102	46/51 and 46/51	1.00 (0.88,1.14)
Drug-related AEs	3	674	50/349 and 54/325	0.80 (0.57,1.14)
DTG vs. EFV	1	113	19/69 and 14/44	0.87 (0.49,1.54)
RAL vs. EFV	2	561	31/280 and 40/281	0.78 (0.50,1.21)
Discontinuation for drugs	3	674	2/349 and 8/325	0.30 (0.08,1.09)
DTG vs. EFV	1	113	0/69 and 2/44	0.13 (0.01,2.62)
RAL vs. EFV	2	561	2/280 and 6/281	0.39 (0.09,1.64)
Grade 3–4 AEs	3	674	78/349 and 87/325	0.89 (0.68,1.15)
DTG vs. EFV	1	113	3/69 and 2/44	0.96 (0.17,5.50)
RAL vs. EFV	2	561	75/280 and 85/281	0.89 (0.68,1.15)
IRIS (Grade 3–4)	3	674	13/349 and 20/325	0.63 (0.32,1.25)
DTG vs. EFV	1	113	1/69 and 1/44	0.64 (0.04,9.93)
RAL vs. EFV	2	561	12/280 and 19/281	0.63 (0.31,1.28)
Death	3	672	13/350 and 16/322	0.80 (0.39,1.64)
DTG vs. EFV	1	113	0/69 and 0/44	NA
RAL vs. EFV	2	559	13/281 and 16/278	0.80 (0.39,1.64)

Since publication bias was hard to detect when the number of studies was small, publication bias was not examined.

## Discussion

This meta-analysis pooled data from 3 trials, showing that in TB/HIV patients, the effects of INIs-based ART was non-inferior to EFV-based ART. Two primary efficacy outcomes (virologic and bacteriology suppression) were effectively controlled. Prespecified subgroup analyses suggested that these results were robust in DTG- and RAL-containing ART. Two rospective cohort studies [43, 44] were excluded from this meta-analysis, owing to their non-RCT nature. In these two studies, it was showed that INIs was associated with favorable TB treatment outcome and viral suppression, which was similar to our study.

We also had several key secondary findings. First, as PLWHA without infection TB [45], INIs had more motivation in terms of CD4^+^ recovery compared with EFV. INIs had the trend of increasing in CD4^+^ cell counts (14.23 cells/μl, 95% CI −6.40, 34.86) when compared with EFV was observed in the present study, but there was no statistically significant difference. This finding was similar to the outcomes in cohort studies [44]. However, in co-infected patients, anti-TB treatment could not restore the immune response, and the HIV-related changes dominate the overall immunological picture [46]. More data are therefore needed to validate our hypothesis. Second, compared with EFV, INIs was not more favorable in improving medication adherence. Though the pill burden and cumulative toxicity could be translated into suboptimal adherence, the two groups both had good adherence at 48 Week. Similar results were also obtained in three antiretrovirals treatment for PLWHA [26]. This might be related to more detailed ART consultation for subjects at the initiation of the trial, which promoted the adherence [47]. Besides, patients whose treatment was discontinued due to drug toxicity were few in our study, since the RIF reduced the concentrations of antiretroviral drugs, which then alleviated the toxic side effects. The effectiveness of adherence also assured treatment satisfaction. It is noteworthy, a recent meta-analysis [48] showed RAL-based dual treatment was more conducive to adherence enhancement in PLWHA, suggesting that simplified treatment  could also contribute to adherence. Yet, there is currently a lack of such studies in the TB/HIV patients. Taken together, of all the efficacy outcomes assessed, none of the EFV-based ART was statistically significantly better than that of the INIs-based ART.

Since more combinations of anti-TB and antiretroviral drugs are being used, the drug toxicity in TB/HIV patients could be of concern. The incidences of AEs in both groups were high, and the main adverse events were serious (grade 3–4). Unexpectedly, the drug-related adverse events were relatively rare. INIs-based ART can achieve fast decrease of the HIV viral load, which gives high expectations regarding immune activation or inflammation recovery. But there is also concern that the drugs may lead to an increased rate of IRIS [49]. Actually, compared to EFV,  a lower incidence of IRIS (grade 3-4) in the INIs group showed that IRIS was uncommon, also indicating the reliability of our findings.

Multiple systematic reviews [50–52] showed that INIs were effective in improving clinical outcomes in PLWHA. Unfortunately, there lacked monitoring data to evaluate the effect of INIs treatment on TB/HIV patients. This meta-analysis provides an important evidence in this regard. In the included studies, INIs only contained DTG and RAL, the two most representative INIs universally recommended as first-line therapy by current guidelines. Both drugs showed similar efficacy [53], which was no inferior to EFV, as shown by the subgroup analyses of this meta-analysis. Furthermore, the subgroup analyses of other efficacy endpoints revealed the same results suggesting that these two drugs are suitable for the co-treatment of TB/HIV patients.

This meta-analysis had a few limitations. Firstly, the number of included studies was very small. We were unable to perform certain subgroup analyses such as the race, since the results have not reported in individual studies. It restricts the generalizability of these results but not their validity. Secondly, patients with ART-experienced were not included in the analysis due to the insufficient study data. We were unable to assess the efficacy in TB/HIV patients who were switched  from an EFV to an INIs regimen. Finally, we could not rule out the possibility of publication bias due to the small number of studies.

## Conclusion

In this meta-analysis, the effects of INIs-based ART are equivalent to EFV-based ART in treatment-naïve of TB/HIV patients. Our finding supports recommending INIs-based ART as first-line treatment in TB/HIV patients, especially under the current guidelines. We hypothesize that INIs-based ART may be more favorable than EFV-based ART, according to the evidence from studies of PLWHA without TB infection. After these ongoing studies [54] are completed and published, we will do further research to validate our hypothesis.

## Supplementary Information

**Additional file 1: Table S1.** Main characteristic of included studies.

**Additional file 2: Fig S1.** Risk of bias graph: Quality assessment about each risk of bias item.

**Additional file 3: Fig S2**. Risk of bias summary: Judgment of the risk of bias for each included study with Cochrane quality assessment tool.

## Data Availability

Data are available from the authors on request.

## Abbreviations

AIDS: Acquired immune deficiency syndrome
AEs: Adverse events
ART: Antiretroviral therapies
CI: Confidence interval
DTG: Dolutegravir
EFV: Efavirenz
HIV: Human immunodeficiency virus
INIs: Integrase inhibitors
IPT: Isoniazid preventive therapy
IQR: Interquartile range
IRIS: Immune reconstitution inflammatory
ITT: Intent-to-treat
NVP: Nevirapine
PIs: Protease Inhibitors
PLWHA: People living with HIV/AIDS
RAL: Raltegravir
RBT: Rifabutin
RCTs: Randomized Controlled Trials
RIF: Rifampicin
RR: Risk ratio
SD: Standard deviation
TB: Tuberculosis
