# Supplementary material for: Integrase inhibitors versus efavirenz combination antiretroviral therapies for TB/HIV coinfection: a meta-analysis of randomized controlled trials
Source: AIDS Res Ther. 2021 May 1;18:25. doi: 10.1186/s12981-021-00348-w (PMC8088572; doi:10.1186/s12981-021-00348-w)
Supplement: Supplementary file 1 — Additional file 1: Table S1. Main characteristic of included studies. [file 12981_2021_348_MOESM1_ESM.doc]

| Study | Methods | ART | Interventions | Cases | Age (medain, IQR, range) | White/Black(%) | Female(%) | HIV-RNA log10 copies/ml (medain,IQR) | Basedline CD4 cells/μl (medain,IQR) |
| --- | --- | --- | --- | --- | --- | --- | --- | --- | --- |
| INSPIRING (NCT02178592) | open, randomised | naive | DTG | 69 | 33(18,62) | 47(68) | 30(43) | 5.1(4.7,5.5) | 208(128,410) |
|  |  |  | EFV | 44 | 32(20,50) | 29(66) | 16(36) | 5.2(4.5,5.7) | 202(92,354) |
| ANRS 12180 (NCT00822315) | multicentre, open, Ⅱ, randomised | naive | RAL | 51 | 37(31,44) | 36(70) | 16(31) | 4.9(4.4,5.4) | 115(50,213) |
|  |  |  | EFV | 51 | 35(29,45) | 35(68) | 12(24) | 5.0(4.5,5.5) | 129(45,308) |
| ANRS 12300 (NCT02273765) | multicentre, open, Ⅲ, randomized | naive | RAL | 230 | 34(28,42) | NA | 92(40) | 5.5(5.0,5.8) | 99(39,239) |
|  |  |  | EFV | 227 | 37(30,43) |  | 90(40) | 5.5(5.0,5.9) | 108(35,238) |
