# Supplementary figures and images for: Integrase inhibitors versus efavirenz combination antiretroviral therapies for TB/HIV coinfection: a meta-analysis of randomized controlled trials
Source: AIDS Res Ther. 2021 May 1;18:25. doi: 10.1186/s12981-021-00348-w (PMC8088572; doi:10.1186/s12981-021-00348-w)

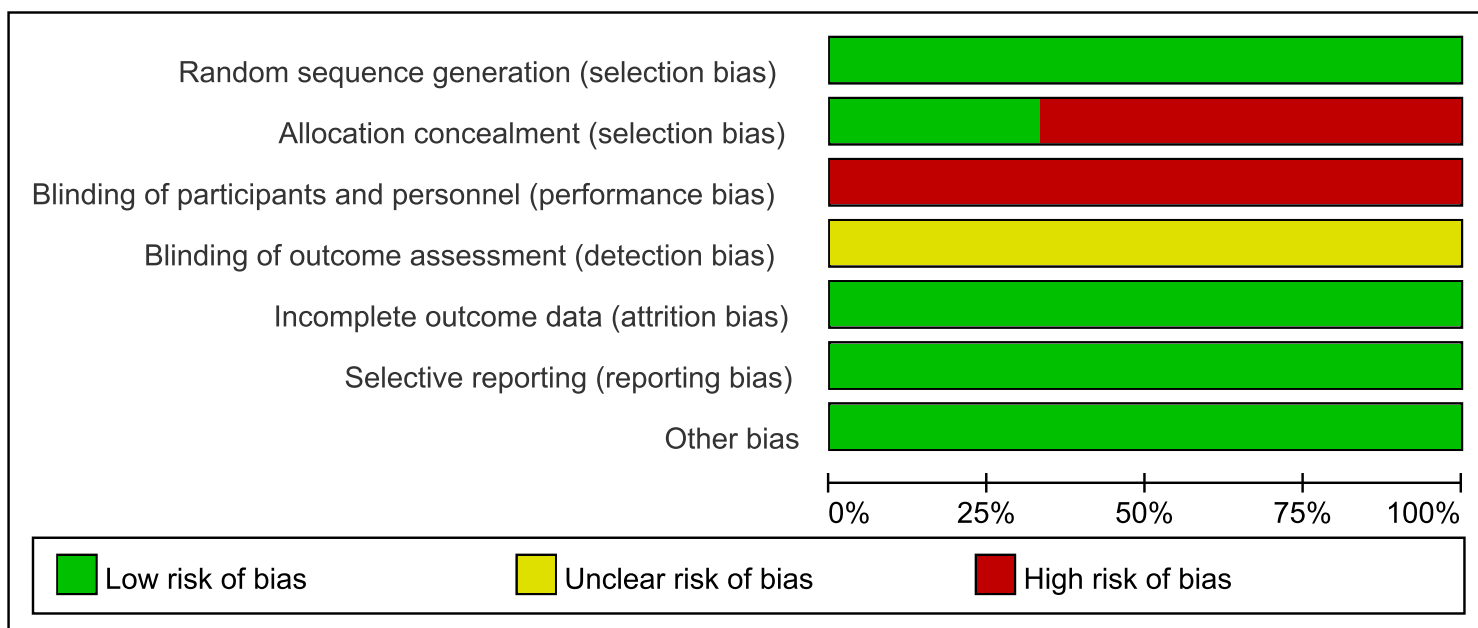

Supplement: Supplementary file 2 — Additional file 2: Fig S1. Risk of bias graph: Quality assessment about each risk of bias item. [file 12981_2021_348_MOESM2_ESM.pdf]
