# Supplementary material for: Integrase inhibitors versus efavirenz combination antiretroviral therapies for TB/HIV coinfection: a meta-analysis of randomized controlled trials
Source: AIDS Res Ther. 2021 May 1;18:25. doi: 10.1186/s12981-021-00348-w (PMC8088572; doi:10.1186/s12981-021-00348-w)
Supplement: Supplementary file 3 — Additional file 3: Fig S2. Risk of bias summary: Judgment of the risk of bias for each included study with Cochrane quality assessment tool. [file 12981_2021_348_MOESM3_ESM.pdf]

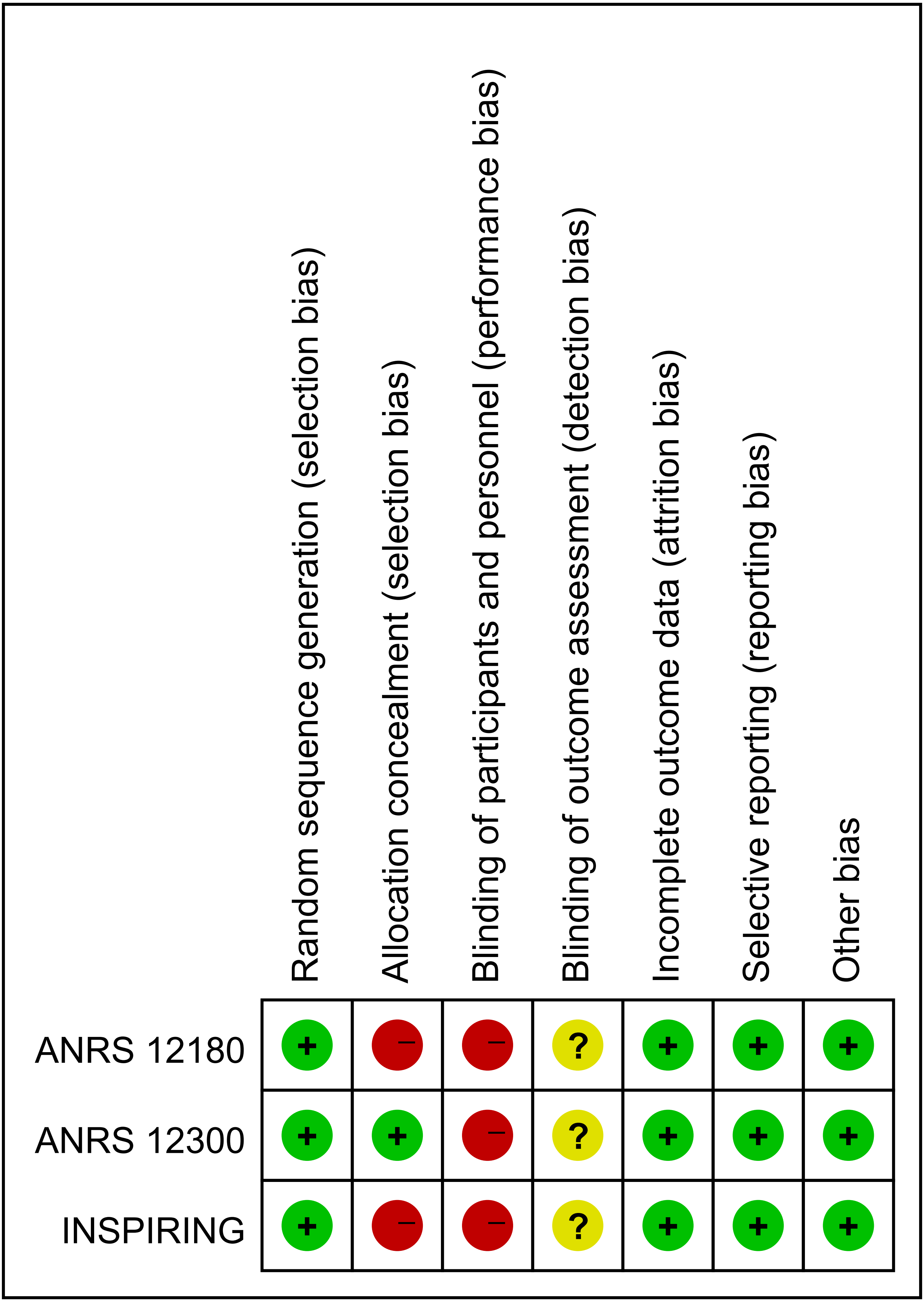
Random sequence generation (selection bias)
Allocation concealment (selection bias)
Blinding of participants and personnel (performance bias)
Blinding of outcome assessment (detection bias)
Incomplete outcome data (attrition bias)
Selective reporting (reporting bias)
Other bias
ANRS 12180
ANRS 12300
INSPIRING
